# Complete Genome Sequence of *Propionibacterium avidum* Strain 44067, Isolated from a Human Skin Abscess

**DOI:** 10.1128/genomeA.00337-13

**Published:** 2013-06-06

**Authors:** Lilla Ördögh, Judit Hunyadkürti, Andrea Vörös, Balázs Horváth, Attila Szűcs, Edit Urbán, Attila Kereszt, Éva Kondorosi, István Nagy

**Affiliations:** Institute of Biochemistry, Biological Research Centre of the Hungarian Academy of Sciences, Szeged, Hungarya; Hungarian Anaerobe Reference Laboratory, Institute of Clinical Microbiology, University of Szeged, Szeged, Hungaryb

## Abstract

*Propionibacterium avidum* is an anaerobic Gram-positive bacterium that forms part of the normal human cutaneous microbiota, colonizing moist areas such as the vestibule of the nose, axilla, and perineum. Here we present the complete genome sequence of *P. avidum* strain 44067, which was isolated from a carbuncle of the trunk.

## GENOME ANNOUNCEMENT

*Propionibacterium avidum* belongs to the ever-growing *Propionibacterium* genus of Gram-positive propionic acid- and acetic acid-producing bacteria. It is considered the third most prevalent species of the genus, after *P. acnes* and *P. granulosum*. While these two species require the presence of skin surface lipids such as sebum, *P. avidum* resides in pilosebaceous follicles of the moist areas of the body such as the axilla, groin, and perineum (1, 2). Although generally considered commensal, it has been isolated from severe infections such as splenic abscess (3), perianal abscess (4), bilateral breast abscess (5), and even from osteomyelitis (6). Despite these reports, *P. avidum* is still a rarely studied organism, with only one partially available genome sequence (oral isolate ATCC 25577, GenBank accession no. AGBA00000000). On this basis, whole-genome sequencing of *P. avidum* strain 44067 was performed to ascertain the relationship of *P. avidum* to other propionibacteria and to facilitate the identification of factors responsible for intraspecies diversity.

Genome sequencing of *P. avidum* strain 44067 was performed by combining the cycled ligation sequencing on a SOLiD V4 system (Life Technologies) with 454 FLX pyrosequencing (Roche). We generated 79,620,962 mate-paired (2- by 25-bp) reads on SOLiD and 175,143 (~369-bp) reads on Roche FLX, which altogether yielded >375-fold coverage. Assembly was performed using GS *de novo* assembler 2.8 and CLC Genomics workbench 6.0.1 provided by 454 Life Sciences and CLC Bio, respectively. Superscaffolding was performed with CodonCode aligner 4.0.3 (Codon Code Corp.) and gap closing was accomplished using PCR followed by Sanger sequencing. Automatic annotation of the genome was performed by the NCBI Prokaryotic Genomes Automatic Annotation Pipeline (PGAAP) (http://www.ncbi.nlm.nih.gov/genomes/static/Pipeline.html), which utilizes GeneMark, Glimmer, and tRNAscan-SE searches. *P. avidum* strain 44067 has a single circular chromosome of 2,526,138 bp, with a GC content of 60.01%. There are 2,297 putative coding sequences, 46 tRNAs, and 9 rRNA loci.

Clustered regularly interspaced short palindromic repeat (CRISPR) loci restrict the acquisition of incoming DNA, thereby limiting horizontal gene transfer (7), and are generally present in the genomes of propionibacteria (8). The CRISPR repeat sequence in *P. avidum* has been previously identified and shown to be present in 28 copies in the genome of reference strain ATCC 25577 (8). The repeat appears in 20 copies in the genome of *P. avidum* strain 44067, suggesting diversity among *P. avidum* isolates. This is further supported by the fact that none of the predicted 18 spacers of the isolate 44067 were found in isolate ATCC 25577. These data raise an exciting possibility that there might be *P. avidum* strains or group of strains with greater potential for opportunistic infection.

### Nucleotide sequence accession number. 

The complete nucleotide sequence of *P. avidum* strain 44067 has been deposited at DDBJ/EMBL/GenBank under accession number CP005287.
